# The fern-feeding genus *Cuprina* Sinev, 1988 (Lepidoptera, Stathmopodidae), new for Taiwan, with descriptions of two new species

**DOI:** 10.3897/zookeys.915.46980

**Published:** 2020-02-24

**Authors:** Zong-Yu Shen, Yu-Feng Hsu

**Affiliations:** 1 Department of Life Science, National Taiwan Normal University, Taipei, 116 Taiwan National Taiwan Normal University Taipei Taiwan

**Keywords:** ferns, host plants, immature biology, spore-feeding

## Abstract

Two new species of Stathmopodidae are described from Taiwan: *Cuprina atayalica* Shen & Hsu, **sp. nov.**, reared from larvae on *Microsorum brachylepis*, and *C. insolita* Hsu & Shen, **sp. nov.**, reared from larvae on *Tectaria subtriphylla* and *T. harlandii*. Diagnostic characters for both species are provided. Larval host plants and the biology of the immature stages of both new species are documented.

## Introduction

The Stathmopodidae (Lepidoptera: Gelechioidea) represent a widespread group of moths which can be recognized by the characteristic rosettes of long and rigid bristles on the segments of the hind leg (Sinev 2015). Species classification of the family is commonly confused mainly due to the lack of diagnostic morphological characters (Hodges 1990). Moreover, some species of this family are difficult to collect, and this problem may contribute to the situation that this group has either been ignored by many lepidopterists or that they are simply rarely encountered.

The collecting efforts of stathmopodid moths mainly relied on light traps, so knowledge is one-sided. We tried to investigate the hostplant associations of stathmopodid moths in Taiwan, using the comprehensive work for the Japanese fauna (Terada 2016) as reference. This strategy has proven effective, with a few unrecorded stathmopodid moths and new hostplant associations discovered during the investigation.

Of the stathmopodid moths discovered from this survey, two species appear to conform to the diagnosis of the genus *Cuprina* Sinev, 1988, which is known from Far East Russia (Sinev 1988), Korea (Koo et al. 2018), Japan (Terada 2016), and Sri Lanka (Meyrick 1913). Larvae of *Cuprina* are known to feed on the spores of ferns (Sinev 1988, Sawamura et al. 2009, Terada 2016). According to the global catalog (Sinev 2015), only two species are included in the genus to date: *C. fuscella* Sinev, 1988 and *C. porphyrantha* (Meyrick, 1913). After comparing the specimens of *Cuprina* collected from Taiwan with both previously known taxa, important differences were noticed in wing pattern and genitalia, and thus we concluded that these two species represent undescribed species. In the present study, these two species are described as new, with information on their immature stages. Accordingly, the species diversity of the genus *Cuprina* has doubled to four species.

## Materials and methods

All adult moths were reared from immature stages collected from their host plants. Genitalia slides were prepared following procedures given by Common (1990). Terminology of genitalia follows Klots (1970) and Koster and Sinev (2003: 59–67), those of wing patterns Koster and Sinev (2003: 59–67). Holotypes will be deposited in the Natural History Museum, London (**NHMUK**). Additional type series or vouchers will be deposited in NHMUK and the Department of Life Science, National Taiwan Normal University, Taipei, Taiwan (**NTNU**).

## Taxonomic accounts

### *Cuprina* Sinev, 1988

*Cuprina* Sinev, 1988: 122. Type species: *Cuprina fuscella* Sinev, 1988, by monotypy.

According to Sinev (1988), species of *Cuprina* can be diagnosed by the following characters: forehead strongly sloping, with smooth-scaled covering; vertex narrow; antenna with rod-shaped basal segment, serrate apically; very short cilia present ventrally; eye-cap not developed; forewing lanceolate; hindwing narrowly lanceolate; color of wing shiny bronzy-purple; metatibia dorsally covered with bristles except near apex; bristles with narrow gap at basal 1/3 of metatibia. Terada (2016) emphasized the bristles on the hind-tibia.

#### Cuprina atayalica

Taxon classificationAnimaliaLepidopteraOecophoridae

Shen & Hsu sp. nov.

1A8778A4-1F34-5922-AF24-376923FEE0C2

http://zoobank.org/C44D6494-3828-4C17-AA31-DC22F693D1F7

**Figures 1–8.**
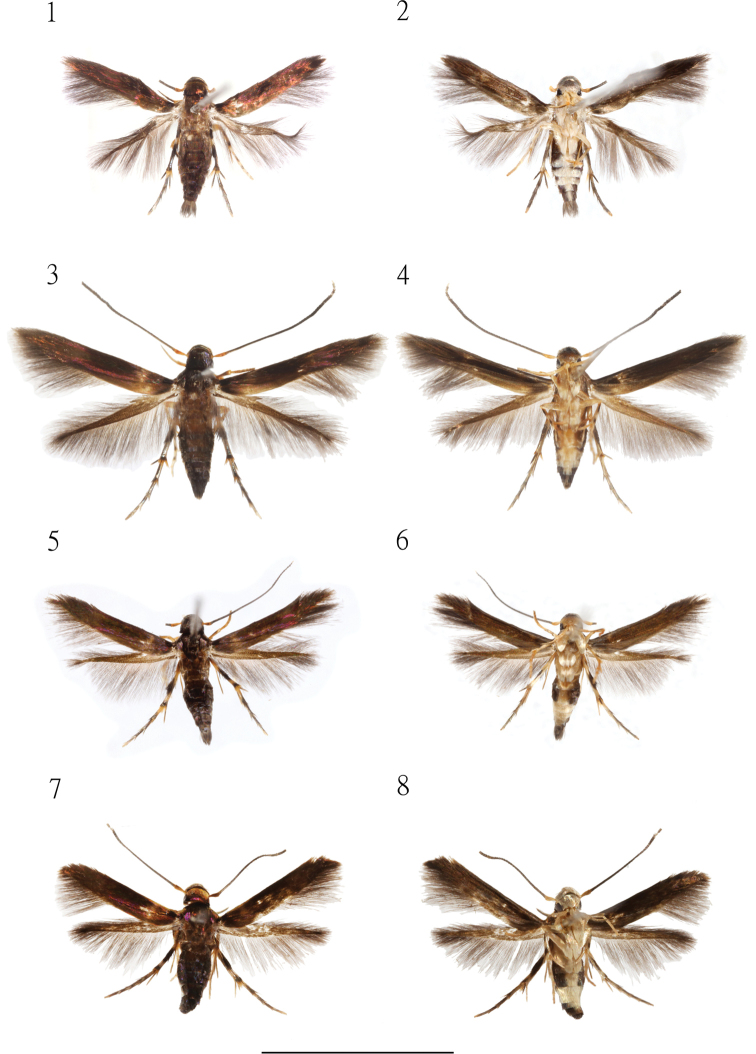
Specimens of *Cuprina* species. **1, 2***C. atayalica* sp. nov., paratype male, Taiwan: New Taipei City, Shuangxi, Yingtzailing **3, 4***C. atayalica* sp. nov., holotype female, Taiwan: Taoyuan, Fuxing, Lalashan **5, 6***C. insolita* sp. nov., paratype male, Taiwan: New Taipei City, Xindian, Hemeishan **7, 8***C. insolita* sp. nov., female holotype, Taiwan: New Taipei City, Xindian, Hemeishan. Scale bar: 5 mm.

**Figures 9–12.**
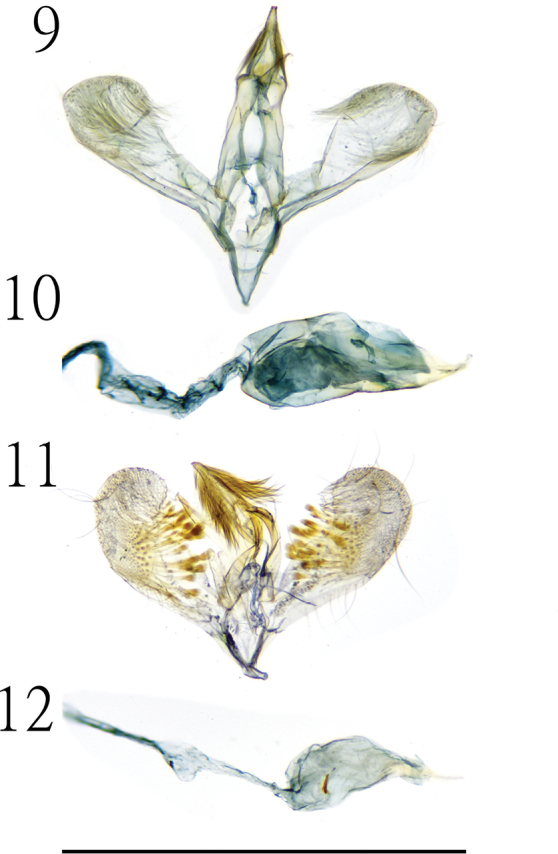
Genitalia of *Cuprina* species **9, 10** male genitalia of *C. atayalica* sp. nov. (Gen. Prep. ZYS-0009, NTNU) **11, 12** male genitalia of *C. insolita* sp. nov. (Gen. Prep. ZYS-0026, NTNU). Scale bar: 1 mm.

**Figures 13, 14.**
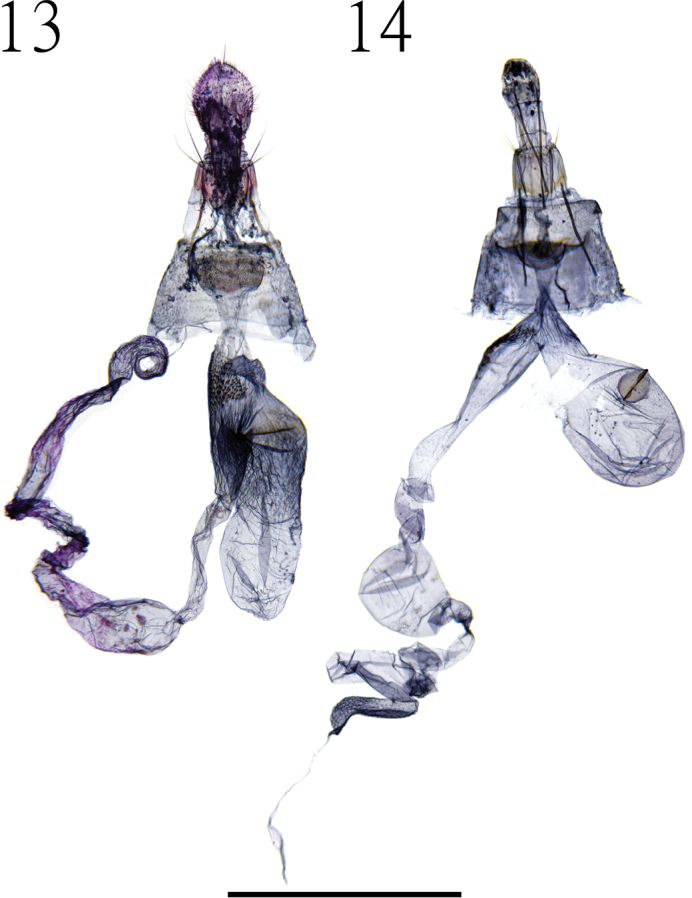
**13** female genitalia of *C.*atayalica sp. nov. (Gen. Prep. ZYS-0090, NTNU) **14** female genitalia of *C. insolita* sp. nov. (Gen. Prep. ZYS-0091, NTNU). Scale bar: 1 mm.

Figs 1–4 , 9–10 , 13

##### Type material.

***Holotype*.** ♀, Taiwan: Taoyuan, Fuxing, Lalashan, ca. 1500 m, 13 Mar 2018, reared from *Microsorum brachylepis*, emg. 22 Apr 2018, Y. F. Hsu, C. W. Huang, C. J. Chang Coll. ***Paratypes*.** 1♂, Taiwan: New Taipei City, Shuangxi, Yingtzailing, ca 1000 m, 29 Dec 2017, reared from *M. brachylepis*, emg. 11 Feb 2018, Z. Y. Shen, Y. Y. Lu, C. J. Chang Coll. (NTNU). 2♂, 3♀, same data as holotype, emg. 9–17 Apr 2018, Y. F. Hsu, C. W. Huang, C. J. Chang Coll. (1♂, Gen. Prep. ZYS-0009, NTNU). 4♀, Taiwan:Taoyuan, Fuxing, Lalashan, ca. 1500 m, 16 Mar 2019, reared from *M. brachylepis*, emg. 19–22 Apr 2019. Z. Y. Shen and G. Y. Chen Coll. (1♀, Gen. Prep. ZYS-0090, NTNU).

##### Diagnosis.

This species can be distinguished from congeneric species by the following characters in genitalia: costa considerably thicker than in the others and basal sclerotized structure in aedeagus not developed. This species can be separated from sympatric *C. insolita* by coloration of abdomen in ventral view: fuscous scales extending ventrad and visible in fourth and fifth abdominal segment in this species, but not extending ventrad and invisible in *C. insolita*.

##### Description.

Male (Figs 1, 2). Forewing length 3.25–3.94 mm (*N* = 3). Head: antenna black, white apically, scape ocherous orange; labial palps slender, long, strongly upcurved, ocherous orange; frons grey, sub-shiny; vertex narrow, bronzy-purple; occiput bronzy-purple, sub-shiny. Thorax: surface covered by shiny bronzy-purple scales. Legs: fore and middle legs shiny grey tinged with white, mesotibia bearing a pair of apical spurs, with outer spur less than 1/2 length of inner spur; hind legs silvery, overlaid with dark brown scaling dorsally; tibia bearing prominent, black, proximal hair tuft and a whirl of orange bristles; tarsus with each tarsomere bearing a whirl of dark brown bristles, metatibia bearing two pairs of spurs, proximal spurs with outer one approximately 1/3 length of inner one, apical spurs with outer one approximately 1/2 length of inner one. Forewing: upperside ground color shiny bronzy-purple with dark brown patch at the 1/3 of costal margin of wing, cilia fuscous; underside uniformly fuscous. Hindwing: ground color uniformly pale fuscous on both sides, cilia fuscous. Abdomen: shiny bronzy-purple dorsally; whitish grey with fuscous lateral markings visible in fourth and fifth abdominal segment from ventral view; anal tuft present.

Female (Figs 3, 4). Forewing length 4.03–4.23 mm (*N* = 5); Similar to male but lacking anal tuft in abdomen.

Male genitalia (Gen. Prep. ZYS-0009, NTNU, Figs 9, 10). Uncus acute-triangular, down-curved apically, with apex truncate; gnathos acute-triangular, slightly longer than uncus, with acute apex; tegument well developed; valva rounded posteriorly; costa thicker than sacculus; costa obvious oblique ventrally; cucullus oval, as long as uncus, with numerous setae on inner surface; saccus approximately 1/2 length of uncus; aedeagus stout, approximately 3× as long as uncus, basal sclerotized structure not development.

Female genitalia (Gen. Prep. ZYS-0090, NTNU, Fig. 13). Apophyses anteriores nearly as long as apophyses posteriores; ostium bursae with prominent sublateral fold, with numerous small spines on inner surface; corpus bursae with large signum, bar-shaped, situated at the middle of corpus bursae, small spines present near the connection of corpus bursae and bulla; bulla assimilated with ductus seminalis; many small spines present at proximal end of ductus seminalis.

##### Etymology.

The specific name is derived from the name Atayal, referring to the indigenous people who live in the region of the type locality.

##### Host plants.

*Microsorum brachylepis* (Baker) T. Nakaike, 1981 (Polypodiaceae)

##### Immatures.

Larva (Fig. 16) vermiform, slender, slightly tapering toward the caudal end; body is creamy white in color, with head capsule and prothoracic shield dark brown, glossy. Pupa (Fig. 17) oval in shape, with head obtuse, attenuate to pointed caudal end.

##### Biology.

**Figures 15–20.**
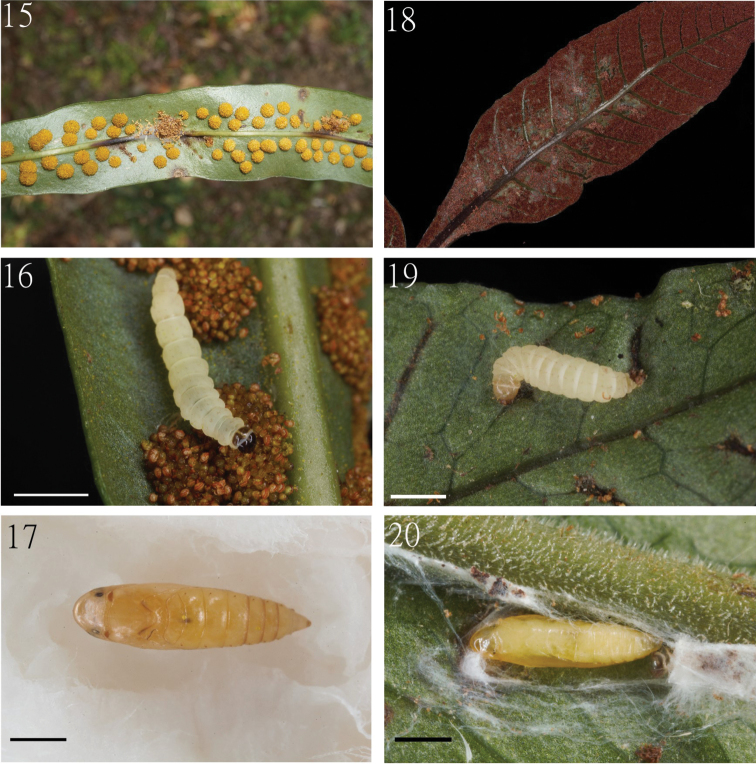
Immatures of *Cuprina* species **15** larval damage by *C. atayalica* sp. nov. on *Microsorum brachylepis***16** larva of *C. atayalica* sp. nov. **17** the pupa of *C. atayalica* sp. nov. **18** larval damage by *C. insolita* sp. nov. on *Tectaria harlandii***19** larva of *C. insolita* sp. nov. **20** pupa and cocoon of *C. insolita* sp. nov. Scale bars: 1 mm.

Larvae were found in January and May. They construct tunnel-like shelters on the underside of the host plant leaves (Fig. 15), composed of fern spores and its own frass. The larvae (Fig. 16) live inside the shelter and feed on the spores until pupation. The pupae (Fig. 17) construct loose cocoons within larval shelters. Adult moths emerged nearly one month after pupation without diapause, suggesting that this species may be multivoltine.

##### Distribution.

Known only from Taiwan.

#### Cuprina insolita

Taxon classificationAnimaliaLepidopteraOecophoridae

Hsu & Shen sp. nov.

9D105C8E-9594-533E-8313-B750376E8C89

http://zoobank.org/86C5B8E0-B001-444E-8612-CD41FF3F0D57

Figs 5–8 , 11–12 , 14

##### Type material.

***Holotype*.** ♀, Taiwan: New Taipei City, Xindian, Hemeishan, ca. 150 m, 29 Apr 2018, reared from *Tectaria harlandii*, emg. 20 May 2018, Z. Y. Shen Coll. ***Paratypes*.** 1♂, 2♀, same data as holotype, emg. 17 May 2018, Z. Y. Shen Coll. (1♂, Gen. Prep. ZYS-0026, NTNU. 1♀, Gen. Prep. ZYS-0091, NTNU). 1♂, 1♀, Taiwan: Taina, Dongshan, Kantoushan, ca. 600 m, 18 Sep 2018, reared from *Tectaria subtriphylla*, emg. 5–6 Oct 2018, Y. F. Hsu Coll. (NTNU).

##### Diagnosis.

This species may be distinguished from other congeneric species by the presence of peculiar specialized clavate setae at the base of the cucullus. The ventral side of the abdomen has the same color pattern as *C. atayalica*, but they can be distinguished by the visibility of fuscous scales in fourth and fifth abdominal segment.

##### Description.

Male (Figs 5, 6). Forewing length 3.07–3.47 mm (*N* = 2). Head: antenna black, white apically, scape ocherous orange; Labial palps slender, long, strongly upcurved, ocherous orange; proboscis covered by maxillary palps; frons grey, sub-shining; vertex narrow, bronzy-purple, sub-shining; occiput bronzy-purple, sub-shining. Thorax: surface covered with shiny bronzy-purple scales.; Legs: fore and middle legs creamy orange, with dark brown patches in mesotibia, mesotibia bearing a pair of apical spurs, with outer spur less than 1/2 length of inner spur; hind legs creamy orange with dark brown patches in metatibia, extensive dark brown scaling on tarsus; tibia bearing prominent black, proximal hair tuft and a whirl of orange bristles; tarsus with each tarsomere bearing a whirl of dark brown bristles, metatibia bearing two pairs of spurs, proximal spurs with outer one approximately 1/3 length of inner one, apical spurs with outer one approximately 1/2 length of inner one. Forewing: upperside ground color shiny bronzy-purple with dark brown patch at 1/3 of costal margin of wing, cilia fuscous; underside uniformly fuscous. Hindwing: ground color uniformly pale fuscous on both sides, cilia fuscous. Abdomen: shiny bronzy-purple dorsally; grey tinged with white, without fuscous lateral markings visible in fourth and fifth abdominal segment from ventral view; an anal tuft present.

Female (Figs 7–8). Forewing length 2.04–3.64 mm (*N* = 4). Similar to male but lacking anal tuft in abdomen.

Male genitalia (Gen. Prep. ZYS-0026, NTNU, Figs 11, 12). Uncus slightly curved apically, with sharp apex, numerous setae present laterally; gnathos narrow, slightly longer than uncus, with truncate apex; tegument well developed; valva rounded posteriorly; sacculus slightly thicker than costa; cucullus oval, longer than uncus with numerous setae on inner surface, a few specialized clavate setae present on cucullus basally; saccus approximately 1/3 length of uncus; aedeagus stout, approximately 1.5× as long as uncus, basal sclerotized structure hook-shaped.

Female genitalia (Gen. Prep. ZYS-0091, NTNU, Fig. 14). Apophyses posteriores longer than apophyses anteriores; ostium bursae with prominent sublateral fold with numerous small spines on inner surface; corpus bursae small with prominent signum, bar-shaped, situated at the middle of corpus bursae; small spines present at the basal of bulla; bulla assimilated with ductus seminalis; many small spines at proximal end of ductus seminalis.

##### Etymology.

The species name is the feminine form of the Latin adjective *insolitus*, for uncommon or unusual, referring to the peculiar clavate setae on the inner surface of the valva not found in any other known stathmopodid moth.

##### Host plants.

*Tectaria subtriphylla* (Hook. & Arn.) Copel., 1907 and *T. harlandii* (Hook.) C. M. Kuo, 2002 (both Tectariaceae).

##### Immatures.

Larva (Fig. 19) vermiform, elongate, somewhat stout, abruptly tapering toward the caudal end; body is creamy white in color, with head capsule and prothoracic shield pale brown, glossy. Pupa (Fig. 20) oval in shape, with head obtuse, attenuate to pointed caudal end.

##### Biology.

Larvae were found in March and September. Larvae associated with *T. harlandii* construct tunnel-liked shelters (Fig. 18) and live gregariously on the underside of the sporophyll. Larvae (Fig. 19) associated with *T. subtriphylla* produce tunnel-liked shelters individually, and also live on the underside of leaves. The pupae (Fig. 20) construct loose cocoons within larval shelters. Larvae were found in spring and autumn; adult moths emerged about one month after pupation, suggesting that the species is multivoltine.

##### Distribution.

Known only from Taiwan.

## Discussion

The larval host plants of *Cuprina atayalica* and *C. insolita* belong to the families Polypodiaceae and Tectariaceae, respectively. These families were previously not recorded as hosts utilized by species of *Cuprina* (Terada 2016). *Cuprina porphyrantha* is known as a specialist on Dryopteridaceae, while *C. fuscella* uses ferns of several families: Woodsiaceae, Onocleaceae, Theyriaceae, and Dryopteridaceae (Sinev 1988, Sawamura et al. 2009, Terada 2016). These fern families all belong to the “eupolypods” clade as defined in the phylogeny by PPG I (2016), so that eupolypods may be the original hostplant association for the genus *Cuprina*. It seems plausible to assume that if additional *Cuprina* moths will be discovered in the future, species of eupolypods will be the most likely hostplants, especially Polypodiineae, since all four known *Cuprina* species use fern families in this group.

## Supplementary Material

XML Treatment for Cuprina atayalica

XML Treatment for Cuprina insolita
